# Use of a white light supercontinuum laser for confocal interference-reflection microscopy

**DOI:** 10.1111/j.1365-2818.2012.03603.x

**Published:** 2012-05

**Authors:** L-D Chiu, L Su, S Reichelt, WB Amos

**Affiliations:** * Light Microscopy Laboratory, Cancer Research UK Cambridge Research InstituteCambridge, U.K.; † MRC Laboratory of Molecular BiologyCambridge, U.K.

**Keywords:** Confocal laser scanning microscopy, interference reflection microscopy, supercontinuum laser

## Abstract

Shortly after its development, the white light supercontinuum laser was applied to confocal scanning microscopy as a more versatile substitute for the multiple monochromatic lasers normally used for the excitation of fluorescence. This light source is now available coupled to commercial confocal fluorescence microscopes. We have evaluated a supercontinuum laser as a source for a different purpose: confocal interferometric imaging of living cells and artificial models by interference reflection. We used light in the range 460–700 nm where this source provides a reasonably flat spectrum, and obtained images free from fringe artefacts caused by the longer coherence length of conventional lasers. We have also obtained images of cytoskeletal detail that is difficult to see with a monochromatic laser.

## Introduction

Interference reflection microscopy (IRM) was introduced by Curtis (1964) as a method for observing the surface of living cells submerged in aqueous medium but in contact with a planar glass substrate. In IRM, light is directed into the back of the objective lens and falls on the specimen. A small portion of this light is reflected back into the objective lens and forms an intermediate image in the normal position in the microscope. Curtis found that regions of close contact between the cell and substrate appeared in such images darker than the background, and interpreted the dark zones as because of destructive interference between light reflected internally in the glass of the cover slip and light reflected off the surface of the cell only a few nanometre away. The interference is destructive because the light that passes from a region of low refractive index (saline or culture medium) and is reflected at the surface with a higher index medium (the cell membrane) undergoes a phase shift of pi or half a wavelength for all frequencies. The light reflected internally at the cover slip water interface is not phase-shifted and is therefore in antiphase to the light reflected off the cell. IRM has been used extensively for studies of cell contacts (see Verschueren, 1985; Izzard & Lochner, 1976, 1980; Beck & Bereiter-Hahn, 1981).

A great improvement in the contrast of IRM images was achieved by the use of confocal scanning optics with laser sources. A clear demonstration of the advantage of combining confocal optics with IRM was the discovery that images could be obtained even of subresolution objects such as individual microtubules (20 nm in diameter), stationary or propelled across the glass substrate by associated motors (Amos & Amos, 1991).

There were two disadvantages in the use of confocal laser scanning microscopes for IRM. One was that because of the high coherence length of the laser, fringes were commonly seen which arose from the far surface of the cell, which was known to be as much as 10 μm distant from the surface of contact. The other was that because the laser was monochromatic, all fringes were identical in appearance, so it was impossible to determine the fringe order and so recognize which fringes arose from the far surface. We hoped that both problems might be mitigated by the use of the supercontinuum source. In the field of Fourier spectroscopy, the fringe pattern arising from a systematic variation in optical path difference is known as an interferogram (see standard optics texts, e.g. Fowles, 1989). According to Fourier theory, if the source spectrum could be made infinitely wide, the interferogram would collapse to an infinitely thin zero-order fringe corresponding to the zero of optical path difference. Our hope was to realize in practice, interference reflection images of living cells in which nothing but the interface between cell membrane and glass was visible. We have therefore constructed an interference reflection microscope equipped with a supercontinuum laser source delivered through and detected in a confocal scan head. The usefulness of the supercontinuum source in scanning laser microscopy has already been demonstrated for confocal fluorescence imaging (Engelhardt & Hoffmann, 2002; McConnell, 2005), for simple reflection imaging (though not interference reflection) by Booth *et al.* (2008) and for noninterferometric confocal imaging spectroscopy (Itzkan *et al.*, 2007). The supercontinuum has also been used for fluorescence lifetime studies (Dunsby *et al.*, 2004).

## Methods

The white light supercontinuum laser was a SC450–4 system prototype kindly donated by Fianium Ltd (Southampton, U.K.). This consisted of a small box (size 25 cm × 30 cm × 15 cm) from which emerged an optical fibre. The emission was 4 W total power, of which approximately 1 W was in the spectral region from 450 to 700 nm. The pulse frequency was 40 ± 0.5 MHz, too high to give any form of patterning in the scanned image, where the pixel dwell time was of the order of 2–8 μs. The laser unit had good short- and long-term power stability.

The average power at the specimen of the supercontinuum was 2 μW (postobjective), and the average laser power of the helium–neon (He-Ne) laser was 1 μW although observing the Newton's rings and fibroblasts. The power of the supercontinuum and the He-Ne laser sources were tuned up for the observation of fish keratocytes and epithelial cells to 5 μW and 28 μW, respectively. The spectrometer used was Ocean Optics USB 4000 (Ocean Optics, Dunedin, FL, U.S.A.).

**Fig. 1:**
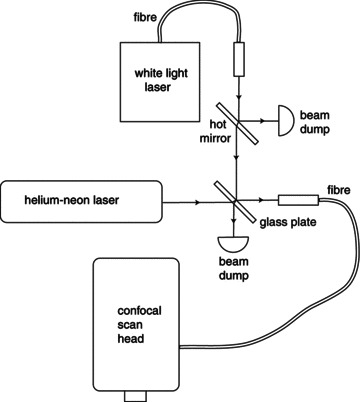
Optical bench allowing the combination of a —He-Ne continuous wave (CW) laser with a white light supercontinuum laser (Fianium, Southampton, U.K.) by a plain glass plate, which allows approximately 90% of the He-Ne beam to pass through and reflects only 5% of the supercontinuum, from which the intense infrared component had already been extracted by means of a hot mirror (Comar Instruments). This method was used to protect the single-mode fibre of the confocal scan head (Bio-Rad Radiance) from excessive supercontinuum power. Because of the poor beam quality of the supercontinuum laser as shown by the size of the best foci obtainable, it was inefficiently coupled in to the single-mode fibre of the scan head and only microwatt powers were available at the sample. This allowed reflection imaging but was insufficient for confocal epifluorescence.

A Bio-Rad Radiance 2000 (Bio-Rad Laboratories Ltd., Hemel Hempstead, U.K.) confocal scanning system with a Nikon Optiphot (Nikon Instech Co., Ltd., Tokyo, Japan) microscope was modified to receive the light from the supercontinuum laser by detaching the single-mode fibre from the built-in lasers of the system (which were not used) and launching the laser light into this fibre (Fig. 1). The fibre was left with its original carefully aligned and cemented connection with the scan head, which was connected to an upright Nikon Optiphot II (Nikon Instech Co., Ltd., Tokyo, Japan) microscope equipped with an epicondenser. To allow nonconfocal IRM observations through the eyepiece, the microscope was equipped with a filter-block unit, in which a 50% reflector could be placed as an alternative to the normal cubes for epifluorescence. When the 50% reflector was introduced, and a halogen lamp attached to the epicondenser, the resulting IRM image could be seen, albeit with low contrast, and proved useful for aligning specimens. For confocal IRM, the reflector was withdrawn and light from the scan head was allowed to pass into the objective and return to the scan head.

The supercontinuum laser was placed on an optical table and the beam was directed into the single-mode fibre leading to the scan head by means of an optical arrangement shown in Figure 1. To safeguard the single-mode fibre, most of the infrared emission from the supercontinuum laser was reflected away by means of a hot mirror plate (Comar Instruments, Cambridge, U.K.) and the remaining radiation was further reduced by allowing most of it to pass through a plain glass plate, which reflected a few percent of the beam into the beam launch optics of the single-mode fibre. The plain glass plate also served as a beam combiner, permitting the use of a red He-Ne laser of 2 mW power to pass through it and so into the single-mode fibre. Although this protection of the single-mode fibre from the power of the supercontinuum may be judged a success insofar as the fibre was not damaged after many hours of continuous use for confocal IRM imaging, it failed to yield high enough powers at the specimen for confocal epifluorescence. Where parallel epifluorescence and IRM observations on the same region of a specimen were required, this had to be done by transferring the specimen to another confocal laser scanning microscope (LSM) and imaging the same region with the aid of a finder grid slide.

For just one initial experiment, as described below, a widefield arrangement was used. The white light laser beam was passed through an opal glass diffuser directly into the epicondenser of the microscope. In this case, instead of the scan head, a conventional digital colour camera was used to record the IRM image. For one observation on IRM with two lasers, a Nikon A1 confocal system (Nikon Instech Co., Ltd., Tokyo, Japan) was used with lasers emitting 571 nm and 637 nm.

## Tissue culture and cell preparation

3T3 fibroblast were cultured on 22 mm diameter cover slips (thickness no. 1.5) in growth medium (Dulbecco's modified Eagle's medium) in 10% fetal calf, 100 IU mL^−1^ penicillin and 100 mg mL^−1^ streptomycin.

### Immunofluorescence staining

The fibroblasts were fixed in absolute methanol at –20°C for 1 min. The cells were then permeabilized with 0.1% Triton in phosphate buffered saline (PBS) for 5 min. Cells were then put in 5% bovine serum albumin (BSA) in PBS for 5 min for preblocking. Actin was labelled with 0.1% phalloidin-TRITC in 5% BSA PBS at 37°C for 1 h. Afterwards, the cover slips were mounted in PBS on a finder slide. Fluorescence images (not shown here) were taken with a Leica TCS SP5 (Leica Mikrosysteme Vetrieb GmbH, Wetzlar, Germany) tandem-scanning system.

### Experiments with a nonliving model

**Fig. 2:**
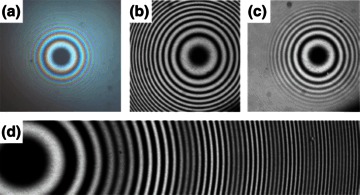
(a) Camera image of Newton's rings with diffused supercontinuum laser source, nonconfocal, (b) scanning confocal image with He-Ne laser, (c) scanning confocal image with supercontinuum source and (d) scanning confocal image of beats pattern obtained with two monochromatic lasers at 531 and 636 nm wavelength. These result show through the fall-off of fringe contrast that the coherence length of the supercontinuum laser is much closer to that of natural light than to that of a monochromatic laser.

Our first experiment with the supercontinuum source used widefield (nonconfocal) microscopy. We set-up a nonliving model specimen for the production of concentric Newton's rings. An objective with a numerical aperture of 0.3 was used to image the area of contact between a convex glass lens with a radius of curvature of approximately 100 mm and a flat glass plate. To fill the back aperture of the lens, the supercontinuum laser light was passed through an opal glass diffuser on its way into the epicondenser. The result (Fig. 2a) was almost identical to that obtained with incoherent natural light (daylight). That is a central dark zero-order fringe surrounded by successively weaker coloured fringes. This proved that the supercontinuum light behaves interferometrically like natural incoherent light with Newton's series of colours.

**Fig. 3:**
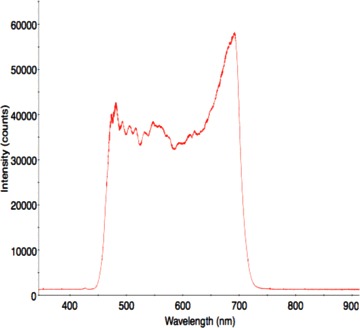
Spectrum of the supercontinuum picked up just before entry to the optical fibre connected to the scan head. Note that the infrared (IR) region is cut-off by the action of the hot mirror. An Ocean Optics USB4000 spectrometer was used, without calibration for absolute intensity. In calibrated spectra supplied by Fianium, this region is flatter and the rise shown here at long wavelength probably reflects the intrinsic response of the silicon detector in the Ocean Optics device.

All subsequent experiments were conducted with scanning optics, using an optical bench (Fig. 1) in which light beams from both a supercontinuum laser and a monochromatic He-Ne laser could be made coaxial, so that either the supercontinuum or the He-Ne laser could be fed via a single-mode optical fibre into a scan head positioned over a conventional microscope. With the glass specimen described above and the same objective, the depth of field was in this case approximately 10 μm, so there was little confocal optical sectioning effect. The image showed a fall-off of fringe contrast in higher order fringes with the supercontinuum source as compared with the monochromatic He-Ne source (Fig. 2b). This difference was expected because of the greater width of the supercontinuum spectrum (Fig. 3).

Keeping with the model specimen, we experimented by interposing a variety of band pass and notch filters between the supercontinuum laser and the single-mode fibre. None of these aided the suppression of higher order fringes. Our conclusion from these experiments was that the bandwidth of the laser was probably limiting the fringe suppression and our manipulations did not increase this. We tested the theory that the radial distribution of intensity in the Newton's rings experiments is the inverse Fourier transform of the spectrum of the source (see e.g. Fowles, 1989) by combining two monochromatic lasers of different wavelength (561 and 636 nm) and imaging in the confocal system as before. As expected, a pattern of beats was obtained (Fig. 2d).

## Observations on living tissue culture cells

**Fig. 4:**
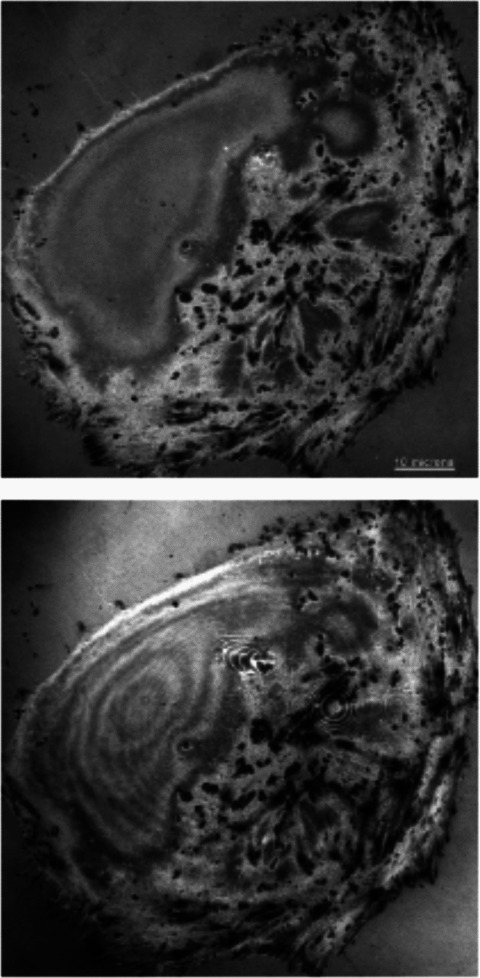
Tissue culture cell, focussed on the interface between the cell and the glass cover slip. The dark features are focal adhesions. The contrast is generated by interference between the light intensity reflected within the cover slip and that which is reflected by the surface of the cell. The latter light is phase shifted by one-half cycle relative to the former. The image obtained with the supercontinuum laser is above, He-Ne below. Note that the perfectly circular artefactual fringes because of the scan lens are absent in the supercontinuum image, as are many of the roughly circular fringes which are believed (from numerous previous studies) to arise from the dorsal (far) dome-shaped surface of the cell.

**Fig. 5:**
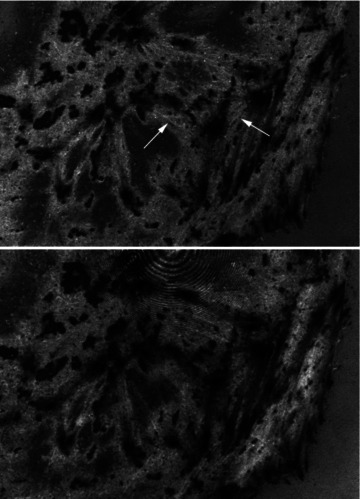
Detail from the images shown in Figure 4. The upper panel shows the supercontinuum image with actin filament bundles (arrowed) which are invisible in the lower image, obtained with a He-Ne laser. However, the He-Ne laser reveals other filament bundles as white lines in the lower region of the figure that are not visible in the supercontinuum image.

Experiments were then performed with living or fixed tissue culture cells. The cells were cultured on glass cover slips and examined using a 100× objective of numerical aperture (N.A.) 1.4 (although, as the cells were mounted in a dilute saline, the effective N.A. was equal to the refractive index of water, approximately 1.3). Under these conditions, the optical sectioning depth is approximately 0.6 μm. A typical image is shown in Figure 4, in which a tissue culture cell is imaged at precisely the same focal level, using first the supercontinuum and then the red He-Ne laser. The most obvious difference is the absence in the supercontinuum image of the intense artefactual circular fringes which arise from reflections in the surfaces of the scanning lens. This well-known artefact had previously been impossible to remove from confocal IRM images. Close to this artefact, it may be impossible to see cytoskeletal structures such as the actin filament bundles shown in Figure 5(b) with the He-Ne laser, whereas they are clear in the supercontinuum image in Figure 5(a). More subtle differences exist, such as the absence of weak fringes in areas of the cell where the interferogram is probably influenced by the cell surface facing away from the cover slip. From previous work, this surface is known to be several microns distant from the near surface in this cell type.

Sets of images were obtained in red, green and blue light by inserting appropriate filters between the glass plate (Fig. 1) and the optical fibre leading to the scan head, or by division of the light in the detector path between the three photomultipliers of the Radiance scan head. Unfortunately, merging these as Red Green Blue (RGB) false colour images gave confusing results, because the different colours were found to focus at different levels. Chromatic aberration could arise from the objective lens, the scan lens and the Kepler telescope in the confocal scan head. We measured this aberration by imaging a reflective aluminium film deposited on the underside of a standard cover slip using the 100× objective and found a focus shift of 0.65 μm when the wavelength was changed from 466 to 613 nm. Consistent coloured images (not shown here) could be obtained only by the time-consuming method of taking three series of images and combining the results from what was judged to be the in-focus image from each series. This was not possible with live cells because movement occurred as the series were being recorded.

## Discussion and conclusions

**Fig. 6:**
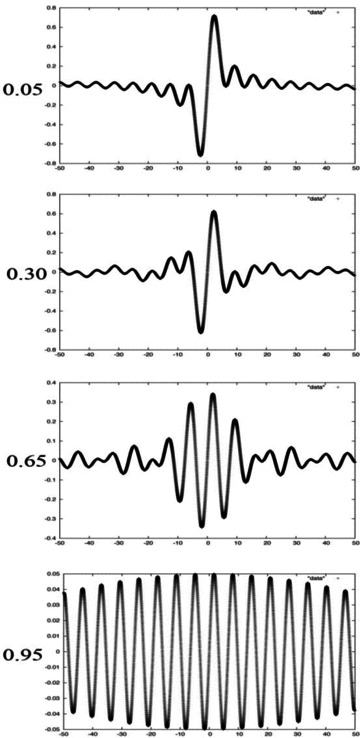
Calculated interferograms in which the intensities of the Fourier transforms of a squarewave spectrum are plotted. The figure to the left of each plot is the ratio of the lowest to the highest wavelength in the input spectrum. The ratio of 0.95 is close to monochromatic, whereas at 0.05 the spectrum spans a 20-fold range of wavelengths.

Although supercontinuum laser source are becoming available for laser scanning microscopes, there seems to have been little comparison with conventional interference reflection. All of our results are consistent with the theory that the pattern of bands in an interference reflection image corresponds to a Fourier interferogram. Having confirmed the applicability of simple Fourier theory, we calculated how, according to theory, the fringe pattern in an interferogram varies with bandwidth between the extremes of bandwidth zero (the monochromatic case) and infinite bandwidth. In Figure 6, bandwidth is expressed as the ratio of the shortest to the longest wavelength in the spectrum. The calculated results show that the observed pattern of fringes is broadly consistent with the supercontinuum bandwidth of 0.66 used in our experiments. It is important to consider also the effect of the bandwidth of the detector. The detectors commonly used in confocal microscopes are photomultipliers, and the bandwidth ratio of the bialkali type is approximately 0.7 (spectral quantum efficiency data from Thorn Vacuum Tubes, Ltd, Electron Tubes Inc., Rockaway, NJ, U.S.A). This suggests that there would be little purpose in using a wider bandwidth from the supercontinuum spectrum unless a different type of detector was used. This small bandwidth explains why we were unable to obtain interference images from which all fringes but the zero-order were eliminated.

In spite of the imperfections of our apparatus, it seems that the white light supercontinuum laser offers definite advantages over conventional lasers in reflection interferometry of living cells. Artefacts from very high-order fringes are eliminated and the clearer supercontinuum images appear in some areas to contain features corresponding to internal cytoskeletal structures not otherwise visible (Figs 4 and 5). However, the visibility of cytoskeletal structures is not always improved by the use of the supercontinuum, perhaps because of the higher contrast in the high-order fringes generated by a monochromatic laser source. Several possible improvements could be made. It would be desirable to increase the efficiency of the transfer of supercontinuum power to the confocal microscope, so that confocal fluorescence (e.g. of known endogenous photoproteins) could be obtained in parallel with the IRM images. The white lines of submicron width in Figure 5 are interpreted above as actin filament bundles because similar fluorescent lines were seen in comparable preparations stained with fluorescent phalloidin examined confocally on a different microscope. With suitable apparatus this could be confirmed by parallel imaging. It would also obviously be an improvement to add to our apparatus a means of temperature control, to improve the viability of the cells under study. The chromatic aberration of the system could be improved by the use of more modern violet-corrected objectives. A more difficult improvement would be to increase, the detection bandwidth which would then justify the use of a broader section of the continuum.

The conventional view that the darkest areas in IRM represent regions of close proximity of the membrane to the cover slip has been challenged by Iwanaga *et al.* (2001), who have introduced a novel and quite different method for determining the size of the gap between the membrane and the substrate. In their method, the membrane is labelled uniformly with a fluorescent dye and the substrate is a highly reflective silicon surface. Interference occurs between light emitted by the dye which passes straight into the objective lens and the part of the emitted light which is reflected by the substrate. These authors, who found by this combination of fluorescence and interferometry no correlation between the dark areas and areas where the gap was narrower, ascribe the dark zones in IRM to the presence of a high concentration of intracellular dry mass within the membrane. This mass concentration seems likely, because the dark zones correlate precisely with the so-called ‘focal adhesions’ which contain high concentrations of intracellular cytoskeletal components.

Our results are presented in the hope that researchers who have confocal microscopes already equipped with supercontinuum sources will be encouraged to use them for interference reflection imagining as well as the excitation of fluorescence. The modification needed is merely the substitution of a semireflecting optical element instead of the chromatic reflector used for fluorescence, though additional advantages may be obtained by the various refinements which have been developed to improve the contrast of widefield (Verschueren, 1985) and which may be applicable also to confocal reflection microscopy (Booth *et al*., 2008). With modern LSMs, equipped with a supercontinuum source, it should be easy to obtain images in confocal epifluorescence precisely registered with the clarified type of interference reflection image demonstrated here. This may well reveal greater detail of processes such as cell adhesion, endocytosis and exocytosis.
